# About a rare cause of a coxitis

**DOI:** 10.1002/ccr3.2578

**Published:** 2019-12-08

**Authors:** Dhia Kaffel, Wafa Triki, Kaouther Maatallah, Hanene Ferjani, Hend Riahi, Zied Jlalia, Wafa Hamdi

**Affiliations:** ^1^ Faculty of Medicine of Tunis University of Tunis El Manar Tunis Tunisia; ^2^ Rheumatology Department Mohamed Kassab National Institute of Orthopaedics La Mannouba Tunisia; ^3^ Radiology Department Mohamed Kassab National Institute of Orthopaedics La Mannouba Tunisia; ^4^ Pediatric orthopaedics Department Mohamed Kassab National Institute of Orthopaedics La Mannouba Tunisia

**Keywords:** acetabulum, coxitis, CT scan, hip, osteoid osteoma

## Abstract

OO of the acetabulum can clinically and radiologically mimic an ankylosing spondylitis (with coxitis and sacroiliitis). Management of OO include multidisciplinary approach including radiologist, rheumatologist, and orthopedist.

## Graphical abstract

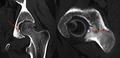

Osteoid osteoma of acetabulum can be challenging to diagnose. A case of an osteoid osteoma of the acetabulum is presented in this article. The lesion mimicked an ankylosing spondylitis. Osteoid osteoma should be included in the differential diagnosis of intractable hip pain.

A 17‐year‐old Tunisian boy without significant medical history presented a 5‐month history of pain in his left hip that was predominantly nocturnal and sensitive to NSAIDS. A clinical examination showed lameness while walking with limitation of hip movements. FABER and sacroiliac compression tests were positive on the left. Inflammatory markers were within the normal range. Pelvic X‐rays were normal. The Ultrasound showed an intra‐articular effusion and a synovitis. The MRI showed an edema of the left acetabulum extending to the left sacroiliac joint. The CT scan (Figure 1) showed a bone sclerosis and a subchondral lytic lesion in the posterior wall of the left acetabulum centered by a calcification related to a nidus of an Osteoid Osteoma (OO).

**Figure 1:**
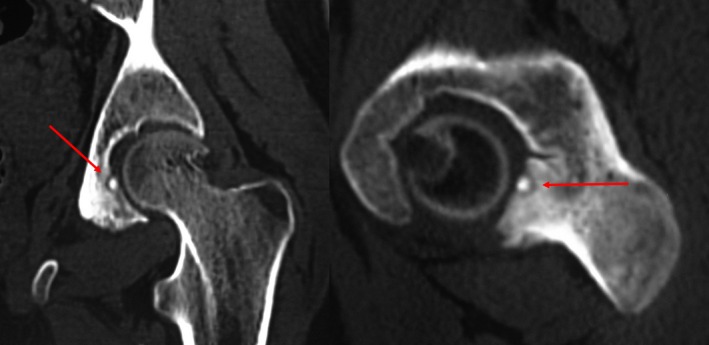
CT scan: bone sclerosis with a subchondral lytic lesion centered by the nidus

The case was discussed in the multidisciplinary staff of the hospital, and the patient has undergone percutaneous ablation under CT guidance (Figure 2). He has a favorable immediate relief of the known pain. No relapse was occurred during 10‐month follow‐up.

**Figure 2:**
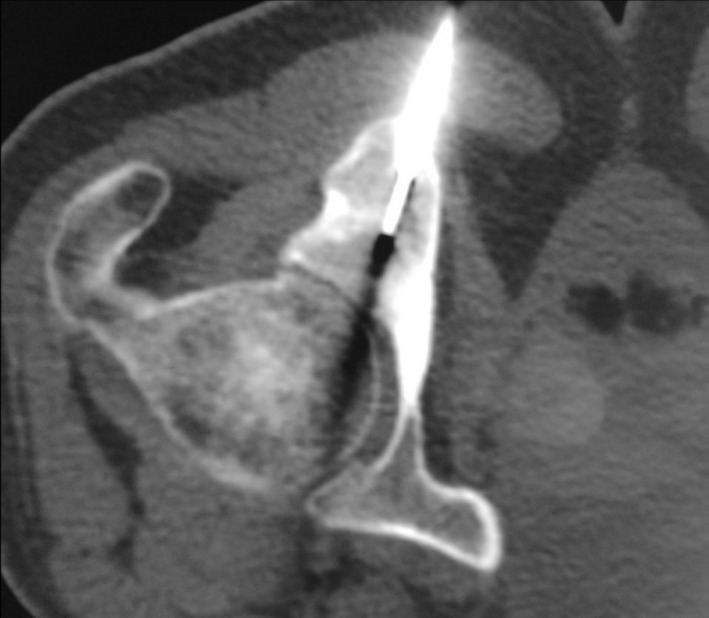
A thin drill is placed in the osteoid osteoma nidus

OO occurs primarily in the lower limbs, and acetabular involvement is very rare (less than 1%).^1^ The important clinical teaching of our case is that OO of the acetabulum can clinically and radiologically mimic an ankylosing spondylitis (with coxitis and sacroiliitis).^2^ The physician should be aware of the potentially confusing clinical and radiographic findings. CT scan is the best imaging technique to diagnose the OO of the acetabulum.

## CONFLICT OF INTEREST

None declared.

## AUTHOR CONTRIBUTIONS

DK: took the pictures with the help of all the coauthors and wrote the text with the help of all co‐authors. WT: submitted this manuscript, was the first doctor who saw the patient, and helped in making the final diagnosis. I also participated in taking pictures and writing the text. KM and HF: helped us to make the final diagnosis and participated in taking pictures and writing the text. HR: helped us to make the final diagnosis, participated in taking pictures and writing the text, and helped with the percutaneous ablation under CT guidance. ZJ: helped us to make the final diagnosis and helped with the percutaneous ablation under CT guidance. WH: supervised all the work. All authors have no potential conflicts of interest, no financial disclosures, and no funding sources.
